# Comparative analysis of bioactivities of leaf extracts from wild plant species *Verbascum sinuatum*, *Amaranthus spinosus*, *Carduus getulus*, and *Heterotheca subaxillaris* collected in Gaza Strip, Palestine

**DOI:** 10.3389/fphar.2026.1753226

**Published:** 2026-02-05

**Authors:** Mohamad Abou Auda, Mohammed Eleyan, Tarek Atia, Hader I. Sakr

**Affiliations:** 1 Department of Biology, Faculty of Applied Science, Al-Aqsa University, Gaza, Palestine; 2 Department of Laboratory Medical Sciences, Al-Aqsa University, Gaza, Palestine; 3 Faculty of Medicine, Al Azhar University Gaza, Gaza, Palestine; 4 Department of Medical Laboratory Sciences, College of Applied Medical Sciences, Prince Sattam Bin Abdulaziz University in Al‐Kharj, Al‐Kharj, Saudi Arabia; 5 Department of Medical Physiology, Kasr Alainy Faculty of Medicine, Cairo University, Cairo, Egypt; 6 Department of Medical Physiology, General Medicine Practice Program, Batterjee Medical College, Jeddah, Saudi Arabia

**Keywords:** *Amaranthus spinosus*, antimicrobial, antioxidant, *Carduus getulus*, *Heterotheca subaxillaris*, *Verbascum sinuatum*

## Abstract

Traditional medicinal plants are valuable sources of bioactive compounds, many acting synergistically and their therapeutic uses are increasingly recognized. In this study, we examined the antimicrobial activity, antioxidant potential, and phytochemical constituents of four traditional medicinal plants (*Verbascum sinuatum*, *Amaranthus spinosus*, *Carduus getulus*, and *Heterotheca subaxillaris*) from the Gaza Strip in Palestine. Hexane extracts of each species were used for phytochemical characterization using Gas Chromatography - Mass Spectrometry (GC-MS). The total phenolic and flavonoid contents (TPC and TFC) were measured, followed by the 2,2-diphenyl-1-picrylhydrazyl (DPPH) assay to examine the antioxidant activity. The antibacterial activity was assessed using the disc diffusion method. GC-MS analysis revealed that each species contained distinct lipophilic compounds. The major classes of components identified in *V. sinuatum* included saturated fatty acid esters, phenolic antioxidants, and a putative alkaloid. *A. spinosus* contained oxygenated monoterpenes, fatty acid derivatives, and aromatic compounds. *C. getulus* was characterized by monoterpenes, diterpenes, fatty acid derivatives, and phenolic antioxidants. *H. subaxillaris* primarily yielded terpenoids, fatty acid esters, and phenolic compounds. Quantitative phytochemical profiling revealed that *H. subaxillaris* exhibited the highest TPC and TFC among the four species. Hexane extracts of *H. subaxillaris*, *A. spinosus*, *V. sinuatum*, and *C. getulus* demonstrated measurable antioxidant activity and inhibitory effect against *Staphylococcus aureus*, *Bacillus cereus*, *Escherichia coli*, and *Pseudomonas aeruginosa*. Among the extracts, *H. subaxillaris* exhibited the strongest antioxidant and antibacterial activities followed, by *V. sinuatum*, *C. getulus*, and *A. spinosus*. These findings highlight the importance of phytochemical profiling in the discovery of new potential bioactive compounds and support future endeavors to isolate valuable metabolites, investigate their biosynthetic processes, and any structure-activity relationships.

## Introduction

The rise of antimicrobial resistance and the prevalence of diseases linked to oxidative stress underscore the need for research into novel natural bioactive compounds derived from medicinal plants. Synergistic interactions among plant-produced secondary metabolites may yield greater therapeutic benefits than those of single compounds (Dubale et al., 2023). Studying the phytochemical composition and bioactivity of traditional medicinal plants is critical for recognizing their medicinal, nutritional, and industrial potential, which has grown markedly in recent years (Selseleh et al., 2020).

*Verbascum sinuatum* L. (Scrophulariaceae) is a Mediterranean herb widely used in traditional medicine due to the bioactive compounds such as iridoids and polyphenols extracted from its aerial parts (Donn et al., 2023). Traditionally, *Amaranthus spinosus* L. (Amaranthaceae/Chenopodiaceae) is also known for its rich content of flavonoids, phenols, terpenoids, tannins, and glycosides (Adegbola et al., 2020; Ruth et al., 2021). Previous studies have reported that *A. spinosus* shows anti-leprotic, anti-diabetic, anti-inflammatory, and antiandrogenic properties. It has been used in the treatment of gastrointestinal disorders, menstrual cramps, and wounds of the skin (Mamuru et al., 2019). The plant also serves as an important feed source for livestock (Abou Auda, 2025).

*A. spinosus* is considered as an invasive weed in pastures of cattle farming but has been recently identified in the reduction of methane Likewise, the Mediterranean genus Carduus (Asteraceae/Compositae) has traditionally been used to treat rheumatism, digestive disorders, and the common cold. Recent ethnopharmacological and phytochemical investigations have shown that Carduus species contain flavonoids, lignans, alkaloids, sterols, and triterpenes (Al-Shammari et al., 2015). Specifically, *Carduus getulus* Pomel has been used in a clinical trial as a hepatoprotective and antimicrobial agent, with its high lipid content playing a role in improving biochemical parameters and antioxidant defenses (Abou Auda, 2023; 2025).

*Heterotheca subaxillaris* is native to the warmer regions of North America and is found across diverse landscapes, from sandy coastlines to arid deserts, where it thrives even in disturbed habitats. It also exhibits allelopathic effects, which have facilitated its spread (Morimoto et al., 2009). Another factor in its widespread occurrence is the release of a strong camphoraceous odor from the aerial parts, caused by high levels of monoterpenoids and sesquiterpenoids that confer resistance against herbivory (Rajasekar et al., 2022).

In addition, the flavonoids and phenolic compounds methylated by *H. subaxillaris* enhance resilience against environmental stresses, allowing it to thrive where conditions are harsh. Some speculate its success in colonizing open, depleted soils may stem partly from allelopathy - the release of allelochemicals inhibiting the establishment of other species (Rajasekar et al., 2022). Notably, the ecological predominance of these secondary metabolites in disrupted niches, such as sand dunes near the coastline, suggests allelopathy plays a role in its displacement of native flora (Morimoto et al., 2009; Sternberg, 2016). This study is the first to investigate the phytochemical and bioactive properties of four wild medicinal plants from the Gaza Strip, Palestine. Therefore, our study aims to evaluate the antibacterial and antioxidant potential of *V. sinuatum*, *A. spinosus*, *C. getulus*, and *H. subaxillaris* by exploring their phytochemical profiles and bioactivities. By linking plant metabolites production with biological activities, we aim to promote the use of these plants as possible sources of novel bioactive compounds.

## Materials and methods

### Collection and identification of plant material

**FIGURE 1:**
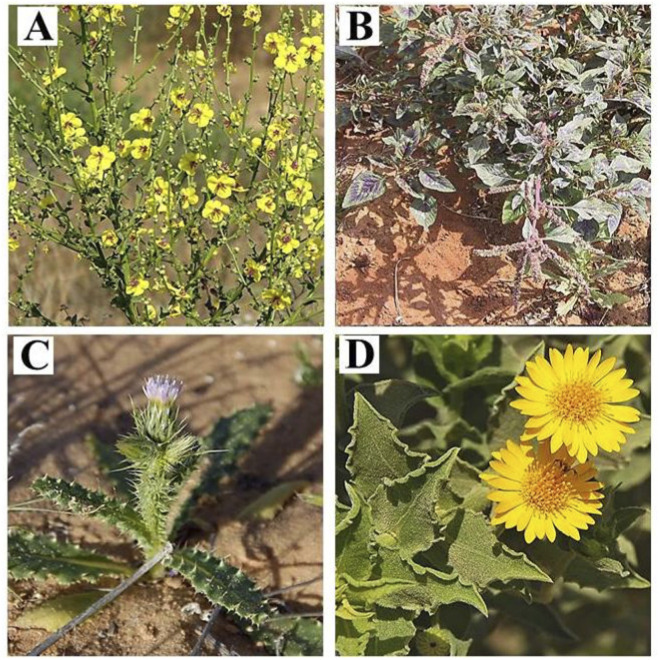
Illustrative photographs of the studied plant species recorded in the Gaza Strip, Palestine: **(A)** *V. sinuatum*; **(B)** *A. spinosus*; **(C)** *C. getulus*; **(D)** *H. subaxillaris*. (https://flora.org.il/en/plants).

Fresh leaves of *V. sinuatum* L., *A. spinosus* L., *C. getulus* Pomel and *H. subaxillaris* (Lam.) Britt. and Rusby (Figure 1) were collected from different locations in the Gaza Strip, Palestine, during the flowering period between March and July 2023. The climate is Mediterranean, with hot summers (temperature of 25 °C–49 °C), mild winters (temperature of 6 °C–13 °C), and annual precipitation ranging from 200 mm in the south to 400 mm in the north per year. Plant species were taxonomically identified by the Botany Department, Al-Aqsa University, Gaza. The leaves of different plants were washed with tap water, then air-dried at room temperature for 10 days, finely ground and stored in a dry environment. Phytochemical analysis were conducted at the Analytical Chemistry and Desert Soils Laboratories of Cairo University, Egypt.

### Extraction of plant material

Ten grams (g) of each sample of leaf powder was extracted in hexane, ethyl acetate and methanol (1:10 w/v) using a Soxhlet apparatus for 6 h for each solvent. Filtrates were concentrated under reduced pressure at 45 °C, evaporated to dryness under a nitrogen stream, and stored at −20 °C in amber vials until analysis (Lee et al., 2016). The crude hexane extract was used for GC–MS profiling, and antioxidant and antimicrobial assays. For colorimetric assays targeting polar constituents (TPC, TFC and DPPH), portions of the dried hexane extracts were subsequently re-dissolved in ethanol to ensure complete solubilization and assay compatibility.

### Gas chromatography-mass spectrometry analysis

The chemical profile was obtained on an Agilent 7000 Triple Quadrupole GC–MS instrument with an Elite-5MS column. The analytical conditions were electron ionization (70 eV), carrier gas helium (1 mL/min, 30:1 split) and temperature ramp 110 °C–280 °C over 36 min. Mass spectra between m/z 45–450 were matched against the NIST library, and compound abundance was calculated from percent peak area using Turbomass software (Ezhilan and Neelamegam, 2012).

### Analysis and characterization of compounds of plant extracts

A combined approach identified the compounds: spectral matching with the mass spectral libraries of NIST (2017) and Wiley (11th edition), calculation of the retention index (RI) in comparison to n-alkanes (C8–C40). Only compounds with >70% agreement were considered confidently identified (Darzi et al., 2025). Quantification was based on individual peak areas (% of total ion chromatogram), processed using Mass Hunter Qualitative Analysis, which provided comprehensive results, including retention times, peak areas, and fragmentation profiles. Triplicate analysis of leaf extracts under identical conditions enabled reproducibility with a relative standard deviation (RSD) of less than 5%. Sample preparation, extract treatment, and GC-MS analysis were combined in one step for data interpretation based on spectral libraries, mass Fragmentation, and RI confirmation.

### Measurement of total phenolic content

Total phenolic compounds in hexane extracts were quantitated by Folin–Ciocalteu assay (Singleton and Rossi, 1965). An aliquot (50–100 mg) of the dried hexane extract was re-dissolved in ethanol to obtain a clear stock solution at 1 mg/mL, as the Folin–Ciocalteu assay requires the analyte to be in a polar medium. A gallic acid stock solution (100,000 ppm) was prepared, and a calibration curve (10–200 ppm) was prepared. To determine, 200 μL of the extract was mixed with 400 μL of the 10% Folin–Ciocalteu reagent, followed by 800 μL of 10% Na_2_CO_3_ after 3 min. Samples, standards, and blanks were stored in the dark for 1 h. The absorbance at 725 nm was measured. The TPC from the standard curve was calculated and expressed as % gallic acid equivalents (%GAE) as follows: TPC (%GAE) = (standard curve concentration/sample weight) × dilution factor × 10,000.

### Measurement of total flavonoid content

Total flavonoids were quantitated according to the aluminum chloride colorimetric assay reported by (Zhishen et al., 1999). Plant material (0.5 g) was homogenized in hexane (10 mL) and filtered. An amount of the dried hexane extract (50–100 mg) was redissolved in ethanol to obtain a clear working solution at a concentration of 1 mg/mL before assaying. A quercetin stock solution (1000 ppm) was prepared (0.1 g in 100 mL), and a working solution (100 ppm) was diluted. A standard curve (20–640 ppm) was created from the standard solutions. For the assay, 125 μL of extract was mixed with NaNO_2_ (75 μL of a 5% solution) and AlCl_3_ (150 μL of a 10% solution), followed by NaOH (750 μL of 1 M) addition, and the volume adjusted to 2.5 mL. Standard solutions and blanks underwent the same treatment. After 15 min in darkness, absorbance was measured at 510 nm. Flavonoid content was calculated as quercetin equivalents (%QE) using a calibration curve and the formula: TFC (%QE) = (standard curve concentration/sample weight) × dilution factor × 10,000.

### DPPH radical scavenging assay

Antioxidant activity of the extracts was assessed using the DPPH (1,1-diphenyl-2-picrylhydrazyl) method on the basis of violet DPPH radical reducibility to the yellow DPPH-H form when interacting with antioxidants. Although hexane was used for initial extraction, the dried crude extract was re-dissolved in ethanol to ensure solubility and assay compatibility. All the tested extracts at various concentrations were examined. In brief, 0.5 mL of DPPH solution (50 mg/100 mL) was mixed with 4.5 mL of hexane, and 0.1 mL of extract was added. The mixtures were shaken and incubated in the dark for 45 min and subsequently assayed for 515 nm absorbance against a blank ((Valko et al., 2007).

### Measurement of Antimicrobial Activity

The antimicrobial activity of hexane crude extracts was evaluated against two Gram-positive bacteria, *Staphylococcus aureus* (methicillin-resistant, MRSA; strain/ID: ATCC 43300) and *Bacillus cereus* (strain/ID: ATCC 14579), and two Gram-negative bacteria, *Escherichia coli* O157:H7 (strain/ID: ATCC 35150) and *Pseudomonas aeruginosa* (strain/ID: ATCC 27853), obtained from the Cairo University bacterial culture collection. Bacterial cultures were grown to logarithmic phase at 37 °C in nutrient broth, adjusted to 1–5 × 10^5^ CFU/mL, and inoculated onto Mueller–Hinton agar plates (agar well diffusion) according to (Hossain et al., 2022). Wells (6 mm diameter) were filled with 100 µL of each extract dissolved in dimethyl sulfoxide (DMSO), with the final DMSO concentration ≤1% (v/v). Plates were incubated at 37 °C for 24 h, and zones of inhibition were measured in millimeters using a digital caliper. Streptomycin (10 μg/mL) served as the positive control, and DMSO (1% v/v) served as the vehicle/negative control.

### Statistical analysis

All the experimental data were analyzed using GraphPad Prism 9.0 (GraphPad Software, San Diego, CA, United States). The normality for the continuous variables was determined using the Shapiro-Wilk test. A one-way ANOVA was performed for parametric data, with Tukey’s *post hoc* test was used for comparisons involving more than two groups. All assays were conducted in triplicate (n = 3) and the data are presented as mean ± SD (standard deviation) from at least three independent experiments. The level of statistical significance was set at *p* < 0.05.

## Results

### GC-MS and phytochemical profiling of *V. sinuatum*

**FIGURE 2:**
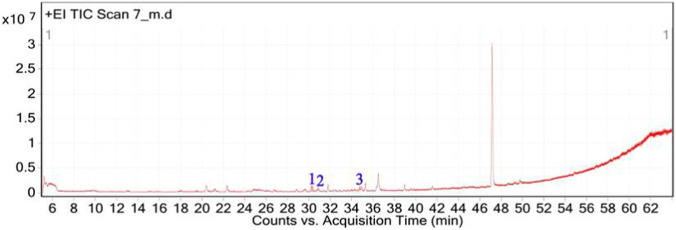
Total ion chromatogram (TIC) of *V. sinuatum* extract with labeled peaks. (1) Methyl palmitate (2) 3,5-ditert-butylphenol, (3) Hordenine.

**FIGURE 3:**
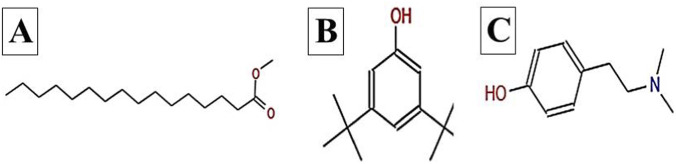
Chemical structures of key compounds identified in *V. sinuatum*: **(A)** Hexadecanoic acid methyl ester **(B)**, **(C)** Hordenine.

The GC–MS chromatogram of *V. sinuatum* revealed three dominant peaks. The most abundant component eluted at 30.243 min and accounted for 65.90% of the total ion current (Table 1; Figure 2). This peak was assigned to methyl palmitate (MW 270.45 g/mol; Figure 3A) based on its EI fragments at *m/z* 74.0 (McLafferty rearrangement) and *m/z* 87.0 (acylium ion), in agreement with NIST library spectra (NIST match above threshold). The second peak (RT 30.840 min; 13.34%) was identified as 3,5-di-tert-butylphenol (MW 206.32 g/mol; Figure 3B). Its EI mass spectrum showed key ions at m/z 191.1 ([M − CH3]^+^), m/z 149 ([M − C4H9]^+^), and m/z 57.1 (C4H9^+^), the tert-butyl–derived alkyl cation formed by cleavage of a tert-butyl substituent. These fragments are consistent with the NIST reference spectrum, and the library search returned match/reverse-match values above the acceptance threshold. A third peak eluted at 34.752 min, accounting for 20.76% of the extract composition. This compound showed spectral similarity to phenethylamine derivatives, specifically a hordenine (C_10_H_15_NO; MW: 165.23 g/mol; Figure 3C), although the match was tentative with a lower confidence score (match score: 54.9). Fragmentation produced characteristic ions at m/z 57, supporting the tentative classification as a phenethylamine-type alkaloid.

**TABLE 1:** Phytochemical constituents of *V. sinuatum* extract characterized by GC-MS analysis.

Peak no.	RT (min)	Area (%)	Chemical compounds	Molecular formula	MW (g/mol)	Key m/z	Match score	Phytochemical class
1	30.243	65.90	Methyl palmitate	C_17_H_34_O_2_	270.45	74.02	71.0	Saturated fatty acid ester
2	30.840	13.34	3,5-Ditert-butylphenol	C_14_H_22_O	206.32	190.57	75.6	Phenolic antioxidant
3	34.752	20.76	Hordenine	C_10_H_15_NO	165.23	190.57	75.6	Alkaloid (phenethylamine)

### GC-MS and phytochemical profiling of *A. spinosus*

**FIGURE 4:**
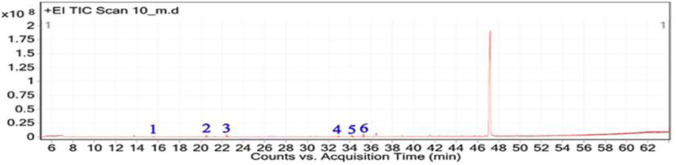
Total ion chromatogram (TIC) of *A, spinosus* extract with labeled peaks. (1) Linalyl acetate (2) 3,7-Dimethyloct-6-en-1-ol, (3) Geraniol (4) 9-Octadecenoic acid, methyl ester, (5) 11,14-Octadecadienoic acid, (6) Isocitronellol.

**FIGURE 5:**
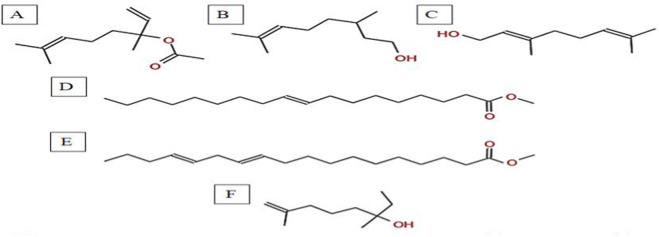
Chemical structures of key compounds identified in *A. spinosus*: **(A)** Linalyl acetate **(B)** 3,7-Dimethyloct-6-en-1-ol, **(C)** Geraniol **(D)** 9-Octadecenoic acid, methyl ester, **(E)** 11,14-Octadecadienoic acid, **(F)** Isocitronellol.

The GC-MS analysis of the hexane extract of *A. spinosus* showed six prominent peaks representing key lipophilic phytochemicals (Table 2; Figures 4, 5). The extract was dominated by monoterpenoid alcohols and fatty acid esters, constituting most of the chemical profile. The most abundant compound, eluting at 35.266 min (Peak 6) and accounting for 30.78% of the total area, was identified as isocitronellol (C_10_H_20_O; MW: 156.27 g/mol). The mass spectrum exhibited a dominant base peak at m/z 82.9, corresponding to [C6H10]+, formed via α-cleavage of the C–O bond in isocitronellol. Despite its moderate match score of 61.4, the compound’s identification is supported by its retention time and co-occurrence with other structurally related terpenes, such as citronellol and geraniol. Citronellol (C_10_H_20_O; MW: 156.27) was identified at 20.510 min (Peak 2; 17.59%), with a match score 76.7. The mass spectrum displayed a characteristic base peak at m/z 69.01, derived from α-cleavage and other ions consistent with the hydroxylated side chain of this acyclic monoterpene. Geraniol (C_10_H_18_O; MW: 154.25), a structural isomer of citronellol, appeared at 22.395 min (Peak 3; 17.34%), with a high match score of 81.4. The spectrum is dominated by m/z 69.00 [C5H9]+, consistent with the α-cleavage of geraniol. Peak 5 (34.160 min; 21.75%) was identified as 11,14-Octadecadienoic acid, methyl ester (C_19_H_34_O_2_; MW: 294.47 g/mol), a polyunsaturated fatty acid ester. Its mass spectrum showed diagnostic ions at m/z 67.02 (due to diene cleavage) and m/z 81.96, typical of linoleic acid derivatives. The high match score of 87.6 supports its confident identification. Methyl elaidate (C_19_H_36_O_2_; MW: 296.49) was identified at 32.923 min (Peak 4; 9.51%), with a match score of 86.9. The compound displayed intense fragment ions at m/z 74.03 (McLafferty rearrangement) and m/z 55.03 (allylic cleavage), considered hallmark ions for mono-unsaturated fatty acid esters. The earliest eluting compound, Peak 1 (15.526 min; 3.03%), was identified as linalyl acetate (C_12_H_20_O_2_; MW: 196.29 g/mol). The mass spectrum exhibited a base peak at m/z 71.01, corresponding to the [C_5_H_11_]^+^ fragment (common for terpenes), along with other diagnostic ions at m/z 92.92 (likely the tropylium ion, C_7_H_7_^+^) and m/z 120.74 (indicative of acetyl cleavage).

**TABLE 2:** Phytochemical constituents of *A. spinosus* extract characterized by GC-MS analysis.

Peak no.	RT (min)	Area (%)	Chemical compounds	Molecular formula	MW (g/mol)	Key m/z	Match score	Phytochemical class
1	15.526	3.03	Linalyl acetate	C_12_H_20_O_2_	196.29	71.01	72.8	Monoterpene ester
2	20.51	17.59	Citronellol	C_10_H_20_O	156.27	69.01	76.7	Monoterpenoid alcohol
3	22.395	17.34	Geraniol	C_10_H_18_O	154.25	92.88	81.4	Monoterpenoid alcohol
4	32.923	9.51	Methyl elaidate	C_19_H_36_O_2_	296.49	55.03	86.9	Fatty acid ester
5	34.16	21.75	11,14-Octadecadienoic acid, methyl ester	C_19_H_34_O_2_	294.47	67.02	87.6	Polyunsaturated fatty acid ester
6	35.266	30.78	Isocitronellol	C_10_H_20_O	156.27	152.77	61.4	Monoterpenoid alcohol

### GC-MS and phytochemical profiling of *C. getulus*

**FIGURE 6:**
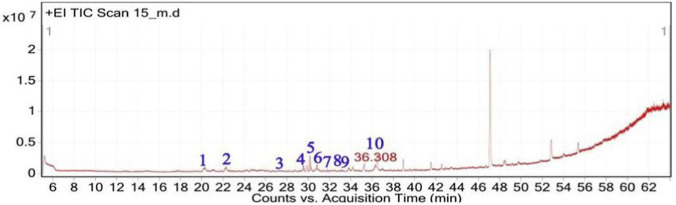
Total ion chromatogram (TIC) of *C. getulus* extract with labeled peaks. (1) Citronellol, (2) Geraniol, (3) Oleic acid (4) Isophytol, (5) Methyl palmitate, (6) 3,5-ditert-butylphenol, (7) Thymol, (8) Arachidonic acid, (9) Methyl undecanoate, (10) Phytol.

**FIGURE 7:**
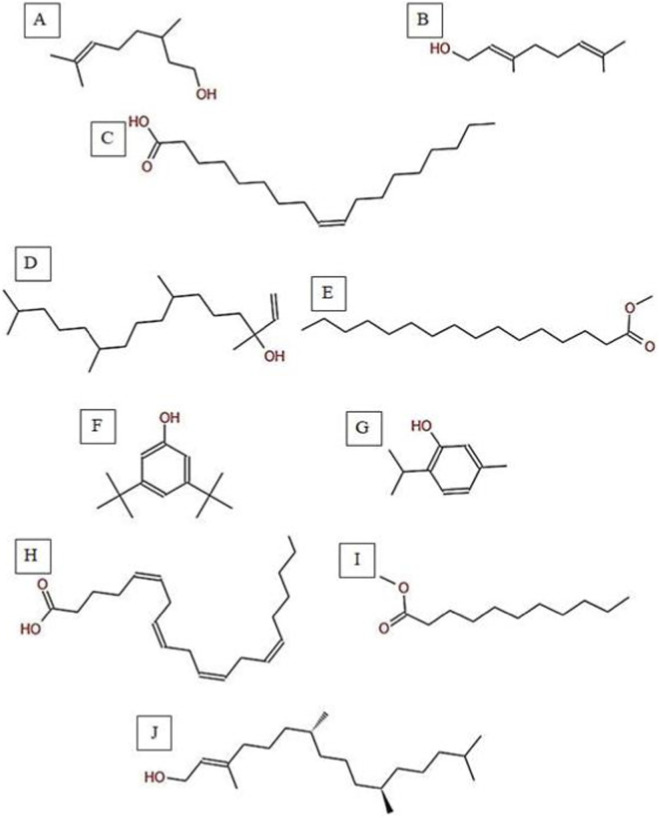
Chemical structures of key compounds identified in *C. getulus*: **(A)** Geraniol, **(B)** Citronellol, **(C)** Oleic acid **(D)** Isophytol **(E)** Methyl palmitate **(F)** 3,5-ditert-butylphenol, **(G)** Thymol **(H)** Arachidonic acid. **(I)** Methyl undecanoate **(J)** Phytol.

The GC-MS analysis of *C. getulus* hexane extract revealed a chemically diverse profile, with ten major peaks corresponding to different classes of lipophilic metabolites (Table 3; Figures 6, 7). The extract was predominantly composed of fatty acid esters, terpenoid alcohols, and phenolic compounds, several of which are known for their pharmacological relevance. The most abundant compound, eluting at 30.127 min (Peak 5) and accounting for 66.26% of the total ion current, was identified as methyl palmitate (C_17_H_34_O_2_; MW: 270.45 g/mol). This saturated fatty acid ester is a common component of plant cuticular waxes and has known antibacterial and anti-inflammatory properties. Its EI mass spectrum exhibited prominent fragment ions at m/z 74.02 (McLafferty rearrangement) and m/z 87.00, supporting its identification with a match score 73.8. 3,5-ditert-butylphenol, a phenolic antioxidant, was the second most abundant metabolite (12.67%; RT 30.800 min; Peak 6). The compound exhibited a high match score of 78.7 and a dominant fragment ion at m/z 190.59, reflecting the loss of a methyl group from the molecular ion. A third principal constituent was isophytol (C_20_H_40_O; MW: 296.5 g/mol), a diterpene alcohol, detected at RT 29.528 min (Peak 4) with 9.58% relative abundance and a base fragment at m/z 57.98. Other notable terpenoids included phytol (C_20_H_40_O; MW: 296.5 g/mol), eluting at 36.308 min (Peak 10; 1.05%), with a prominent fragment at m/z 70.92 and a match score of 76.1. Additionally, thymol, a monoterpenoid phenol, was identified at RT 31.776 min (Peak 7; 4.41%), characterized by m/z 134.76 and a match score of 74.1. Monoterpenoid alcohols were represented by citronellol (Peak 1; RT 20.292 min; 0.88%; m/z 68.98; match score 74.6) and Geraniol (Peak 2; RT 22.135 min; 1.29%; m/z 92.90; match score 77.6). The extract also contained unsaturated and polyunsaturated fatty acids, including oleic acid (Peak 3; RT 27.226 min; 0.69%; m/z 137.84; match score 65.3) and arachidonic acid (Peak 8; RT 33.083 min; 2.14%; m/z 78.85, match score 77.8). Undecanoic acid methyl ester (Peak 9; RT 33.798 min; 1.03%) was identified as a medium-chain fatty acid ester, with a central ion at m/z 73.94 and a match score of 64.6.

**TABLE 3:** Phytochemical constituents of *C. getulus* extract characterized by GC-MS analysis.

Peak no.	RT (min)	Area (%)	Chemical compounds	Molecular formula	MW (g/mol)	Key m/z	Match score	Phytochemical class
1	20.292	0.88	Citronellol	C_10_H_20_O	156.26	69.01	74.6	Monoterpenoid alcohol
2	22.135	1.29	Geraniol	C_10_H_18_O	154.25	92.90	77.6	Monoterpenoid alcohol
3	27.226	0.69	Oleic acid	C_18_H_34_O_2_	282.5	137.84	65.3	Unsaturated fatty acid
4	29.528	9.58	Isophytol	C_20_H_40_O	296.5	57.98	70.3	Diterpene alcohol
5	30.127	66.26	Methyl palmitate	C_17_H_34_O_2_	270.45	74.02	73.8	Saturated fatty acid ester
6	30.800	12.67	3,5-Ditert-butylphenol	C_14_H_22_O	206.32	190.59	78.7	Phenolic antioxidant
7	31.776	4.41	Thymol	C_10_H_14_O	150.22	134.76	74.1	Monoterpenoid phenol
8	33.083	2.14	Arachidonic acid	C_20_H_32_O_2_	304.5	78.85	77.8	PUFA
9	33.798	1.03	Methyl undecanoate	C_12_H_24_O_2_	200.32	74.98	64.6	Fatty acid ester
10	36.308	1.05	Phytol	C_20_H_40_O	296.5	70.92	76.1	Diterpene alcohol

### GC-MS and phytochemical profiling of *H. subaxillaris*

**FIGURE 8:**
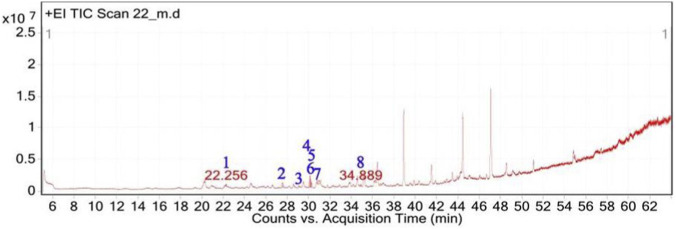
Total ion chromatogram (TIC) of *H. subaxillaris* extract with labeled peaks. (1) Geraniol, (2) Cubebol (3) Falcarinol, (4) Methyl 8-methyl-nonanoate, (5) Methyl palmitate, (6) methyl octadeca-13,16-diynoate, (7) 3,5-ditert-butylphenol, (8) 2,5-Octadecadiynoic acid, methyl ester.

**FIGURE 9:**
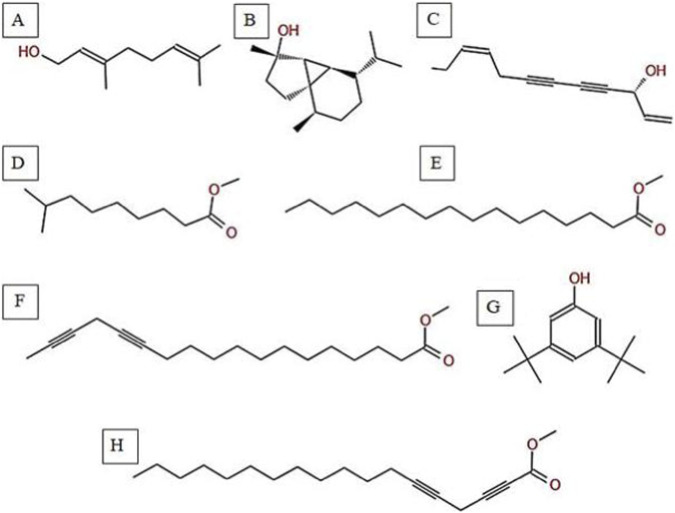
Chemical structures of key compounds identified in *H. subaxillaris*: **(A)** Geraniol, **(B)** Cubebol, **(C)** Falcarinol, **(D)** Methyl 8-methyl-nonanoate, **(E)** Methyl palmitate, **(F)** Methyl octadeca-13,16-diynoate, **(G)** 3,5-di-tert-butylphenol, **(H)** 2,5-Octadecadiynoic acid, methyl ester.

The GC-MS analysis of *H. subaxillaris* hexane extract revealed a chemically diverse and bioactive phytochemical profile consisting of eight major peaks, dominated by fatty acid esters, terpenoid alcohols, polyacetylenes, and phenolic antioxidants (Table 4; Figures 8, 9). The most abundant compound, eluting at 30.130 min (Peak 4), was identified as methyl 8-methyl-nonanoate, constituting 36.84% of the total peak area. The EI mass spectrum exhibited a dominant McLafferty rearrangement fragment at m/z 74.03, confirming its identity as a saturated fatty acid methyl ester with a match score 74.3. Methyl palmitate (C_17_H_34_O_2_; MW: 270.5 g/mol) was the second most abundant metabolite (18.03%, RT 30.153 min; Peak 5), showing a characteristic base ion at m/z 74.02 and a match score of 71.9. Among the early-eluting volatile terpenoids, Geraniol (Peak 1; RT 22.256 min; 3.22%) was identified with a high match score of 78.6. It exhibited key fragment ions at m/z 69.02 (from α-cleavage) and m/z 92.87 (tropylium ion). Cubebol (Peak 2; RT 27.538 min; 18.52%). The spectrum is dominated by m/z 118.76 [C8H14O]+, indicating vinyl ether cleavage in geranyl vinyl ether and a strong match score (78.0). Falcarinol (Peak 3; RT 29.072 min; 3.56%), a long-chain fatty alcohol with a polyacetylene moiety, was confirmed by ions at m/z 128.67, a typical fragment of polyyne cleavage. A unique class of polyunsaturated fatty acid esters was represented by 13,16-octadecadienoic acid methyl ester (Peak 6; RT 30.242 min; 4.31%). The spectrum exhibits a base peak at m/z 74.08 [C3H6O2]+, consistent with McLafferty rearrangement of the methyl ester group in 13,16-octadecadienoic acid methyl ester. Additional peaks at m/z 86.92 [C5H10O]+ and hydrocarbon fragments (m/z 55–69) support the assignment. 2,5-octadecadiynoic acid methyl ester (Peak 8; RT 34.889 min; 6.24%). The spectrum is dominated by m/z 83.95 [C5H7O]+, indicating cleavage near the diyne group. 3,5-ditert-butylphenol (Peak 7; RT 30.772 min; 9.28%) is a well-documented phenolic antioxidant, identified by its base peak at m/z 190.65.

**TABLE 4:** Phytochemical constituents of *H. subaxillaris* extract characterized by GC-MS analysis.

Peak no.	RT (min)	Area (%)	Chemical compounds	Molecular formula	MW (g/mol)	Key m/z	Match score	Phytochemical class
1	22.256	3.22	Geraniol	C_10_H_18_O	154.25	69.03	78.6	Monoterpenoid alcohol
2	27.538	18.52	Cubebol	C_15_H_26_O	222.37	81.87	78.0	Sesquiterpenoid alcohol
3	29.072	3.56	Falcarinol	C_17_H_24_O	244.38	114.75	80.1	Long-chain fatty alcohol
4	30.153	36.84	Methyl 8-methyl-nonanoate	C_11_H_22_O_2_	186.29	74.02	74.3	Branched fatty acid ester
5	30.130	18.03	Methyl palmitate	C_17_H_34_O_2_	270.5	114.75	71.9	Fatty acid methyl ester
6	30.242	4.31	Methyl octadeca-13,16-diynoate	C_19_H_30_O_2_	290.45	190.59	78.7	Polyunsaturated fatty acid ester
7	30.772	9.28	3,5-Ditert-butylphenol	C_14_H_22_O	206.32	55.04	72.9	Phenolic antioxidant
8	34.889	6.24	2,5-Octadecadiynoic acid, methyl ester	C_19_H_30_O_2_	290.45	190.59	78.7	Polyunsaturated fatty acid ester

### Antibacterial activity analysis

The hexane extracts differed statistically significantly (p < 0.05) in inhibition zones within each pathogen (Table 5). *H. subaxillaris* showed a statistically significant (p < 0.05) higher ability to inhibit *Bacillus cereus* than all other extracts, while *V. sinuatum* and *C. getulus* did not show any statistically significant (p > 0.05) difference when compared to each other; however, both were statistically significantly (p < 0.05) more potent than *A. spinosus*.

**TABLE 5:** Antibacterial activity of hexane crude extracts from four medicinal plants.

*Bacterial strain/Plant species*	*V. sinuatum*	*A. spinosus*	*C. getulus*	*H. subaxillaris*
*B. Cereus*	15.2 ± 1.5^b^	8.3 ± 0.6^c^	13.5 ± 1.2^b^	19.8 ± 0.8^a^
*S. Aureus*	12.6 ± 0.9^a^	7.0 ± 1.1^b^	11.8 ± 0.9^a^	12.5 ± 0.7^a^
*E. Coli*	9.3 ± 0.7^a^	5.5 ± 0.8^b^	6.2 ± 0.5^b^	10.1 ± 0.3^a^
*P. Aeruginosa*	7.0 ± 0.5^a^	4.2 ± 0.6^b^	5.0 ± 0.4^b^	8.4 ± 0.9^a^

Mean values (inhibition zone, mm) labeled with different superscript letters (a–c) are significantly different (p < 0.05), according to Tukey’s HSD, test. SD: standard deviation of triplicate experiments.

For *Staphylococcus aureus* (MRSA), *V. sinuatum*, *C. getulus*, and *H. subaxillaris* did not show any statistically significant (p > 0.05) difference compared to one another, but each was statistically significantly (p < 0.05) more potent than A. spinosus.

Regarding *Escherichia coli* and *Pseudomonas aeruginosa*, *V. sinuatum* and *H. subaxillaris* did not show any statistically significant (p > 0.05) difference. They were statistically significantly (p < 0.05) more porent than *C. getulus* and A. spinosus. The latter two extracts did not differ statistically significantly (p > 0.05) from each other (Table 5).

### Assessment of antioxidant capacities in plant extracts

**FIGURE 10:**
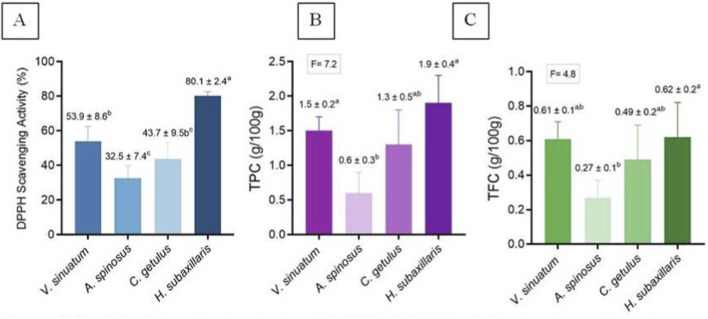
**(A)** Antioxidant activity; **(B)** TPC; **(C)** TFC of *V. sinuatum*, *A. spinosus*, *C. getulus*, and *H. subaxillaris* hexane extracts. Values are presented as mean ± standard deviation (n = 3, from independent experiments). Various lowercase letters indicate significant differences between plants according to one-way ANOVA followed by Tukey’s HSD *post hoc* test (p < 0.05).

Antioxidant activity, in the form of DPPH radical-scavenging, among species were discussed in (Figure 10A). *H. subaxillaris* showed a significantly higher capacity (p < 0.05) than all other plants. Moreover, *V. sinuatum* was statistically significantly (p < 0.05) higher than *A. spinosus* and *C. getulus*, both of which did not reveal any statistically significant (p > 0.05) difference when compared to each other.

### Quantification of bioactive compounds

TPC is presented in Figure 10B. *H. subaxillaris* and *V. sinuatum* were statistically significantly (p < 0.05) higher than *A. spinosus* and *C. getulus.* When compared to each other, neither *H. subaxillaris* and *V. sinuatum,* nor *A. spinosus* and *C. getulus* showed any statistically significantly (p > 0.05) differences. In terms of TFC, the only statistically significant (p < 0.05) was noted between *H. subaxillaris* and *A. spinosus.*).

## Discussion

Medicinal herbs are increasingly recognized as vital sources of alternative pharmacological agents, particularly in the context of rising antibiotic resistance and the prevalence of oxidative stress-related diseases (Zouine et al., 2024). This study investigated the antioxidant and antimicrobial properties, alongside the phytochemical content, of hexane crude extracts from *Verbascum sinuatum*, *Amaranthus spinosus*, *Carduus getulus*, and *Heterotheca subaxillaris*.

This study provides the first report from the Gaza Strip, utilizing comparative GC–MS profiling across species to detect rare lipophilic metabolites and explore specific bioactivity correlations. For GC-MS profiling of *V. sinuatum* hexane extract revealed a phytochemical profile dominated by bioactive and lipophilic metabolites, consistent withits traditional medicinal use and recent evidence of its biological potential. The extract primarily contained hexadecanoic acid methyl ester, 3,5-bis(1,1-dimethyl ethyl) phenol, and a putative hordenine. Hexadecanoic acid methyl ester, the most abundant compound, has been reported in previous studies to exhibit antibacterial, antioxidant, hypocholesterolemic, nematicidal, pesticidal, lubricating, hemolytic, and antiandrogenic activities (Gupta et al., 2023). In line with previous observations reported in the literatures, the lipophilicity of this compound has been suggested to facilitate penetration into Gram-positive bacteria (Shaaban et al., 2021). The free radical scavenging activity observed in the DPPH assay is likely due to the phenolic antioxidant 3,5-bis(1,1-dimethylethyl)-, a sterically hindered compound. Since it contains sterically hindered tert-butyl groups that are resistant to oxidative degradation, this molecule belongs to the family of tert-butylphenol (TBP) antioxidants, a group known for its high stability and strong antioxidant activity (Hoang and Park, 2024).

Despite its low concentrations, 3,5-di-tert-butylphenol is important as it has been shown in previous studies to inhibit acid production and biofilm formation of *Streptococcus mutans* (Vijayakumar and MuhilVannan, 2021). Due to its stability, tert-butylphenol (TBP), a phenolic ring with a tert-butyl substituent, is often used as an antioxidant (Eleyan et al., 2024).

The compound is tentatively identified as hordenine, a phenethylamine derivative. Hordenine and other phenethylamine-related alkaloids are well documented to possess bioactive (Su et al., 2022). Neuroactive, antibacterial properties (Xu et al., 2023; Zhang et al., 2021).

Phenolic antioxidants and lipid-soluble fatty acid esters in *V. sinuatum* may represent an adaptation to abiotic stress factors such as oxidative stress or UV radiation in semi-arid and arid environments. In contrast to other species studied here, such as *H. subaxillaris*, which accumulate methylated polyacetylenes and sesquiterpenoids as diagnostic metabolites, *V. sinuatum* is instead characterized by its abundance of lipophilic esters and phenols. As a consequence of its distinct phytochemical profile may contribute to multi-target bioactivity and could play a role in enhancing defense mechanisms against oxidative stress and infection, as suggested by previous reports (Donn et al., 2023; Saini et al., 2024). Focus on synergistic activity of mixtures of chemical classes in crude extracts, not individual compound treatments, is in line with new trends in natural product research.

The hexane extract of *A. spinosus* contained a wide range of lipophilic components, mainly oxygenated monoterpenes, aromatics, and fatty acid derivatives. The notable components were geraniol, 3,7-dimethyloct-6-en-1-ol, linalyl acetate, 9-octadecenoic acid methyl ester, 11,14-octadecadienoic acid methyl ester, and isocitronellol. The most prevalent components were geraniol, linalyl acetate, and isocitronellol (tentative identification). The antibacterial properties of this extract may be attributed to its oxygenated monoterpenes, citronellol and geraniol, both widely documented for their membrane-dissolving effects in bacteria (Chen and Viljoen, 2022; Jayaraj et al., 2022). Isocitronellol was tentatively identified with a low match score and requires further confirmation. The antibacterial activity of *A. spinosus*, as demonstrated in bioassays against MRSA and *E. coli* O157:H7, is consistent with earlier reports but is comparatively weaker. This may be partly attributed to fatty acid derivatives such as methyl 9-octadecenoate and methyl 11,14-octadecadienoate, which increase lipophilicity and may facilitate membrane permeability, although their exact role requires further investigation (Hartman et al., 2025; Guimarães and Venâncio, 2022).

The *C. getulus* hexane extract contained a mixture of various lipophilic components, identified as monoterpenoids (citronellol, geraniol), diterpenoids (phytol, isophytol), fatty acid derivatives (methyl palmitate, oleic acid, arachidonic acid, methyl undecanoate), and phenolics (3,5-di-tert-butylphenol, thymol). Methyl palmitate was confidently identified with a match score of 73.8 and accounted for 66.26% of the total chromatogram, while 3,5-di-tert-butylphenol, with a match score of 78.7, accounted for 12.67%. These two compounds were confidently identified, whereas methyl undecanoate and oleic acid, with match scores of 64.6 and 65.3 respectively, were provisionally identified and require further confirmation.

This composition suggests potential antioxidant properties, particularly from the confidently identified 3,5-di-tert-butylphenol (Masyita et al., 2022) and possible synergistic antimicrobial effects involving compounds such as geraniol and citronellol (Guadie et al., 2020; Kasmi et al., 2021; Sytar et al., 2018; Tlais et al., 2023). However, the biological relevance of compounds identified with lower match scores, such as methyl undecanoate and oleic acid, should be interpreted with caution until further validation is conducted. Additionally, the diverse composition may indicate a dual ecological role in both direct pathogen inhibition and plant defense signaling (Chauhan et al., 2017), but further investigation is needed to confirm the ecological significance of these compounds.

Our results showed that the hexane extract of *H. subaxillaris* exhibited a chemically diverse and biologically active phytochemical profile, comprising fatty acid esters, terpenoid alcohols, polyacetylenes, and phenolic antioxidants. Key phytoconstituents identified included methyl 8-methyl-nonanoate, methyl palmitate, geraniol, cubebol, falcarinol, 13,16-octadecadienoic acid methyl ester, 2,5-octadecadiynoic acid methyl ester, and 3,5-di-tert-butylphenol. Among these, the identities of methyl 8-methyl-nonanoate, methyl palmitate, geraniol, and cubebol were confirmed with high confidence, based on their relatively higher match scores.

The extract is notable for its high terpenoid content and the presence of rare fatty acetylenic esters. Monoterpenes, sesquiterpenes, including geraniol and cubebol, have been reported in previous studies to be emitted from aerial plant parts and to play roles in plant defense systems (Lange, 2015). Numerous terpenes and terpenoids structurally related to those detected in this extract have been isolated or synthesized and reported to show potential chemotherapeutic properties, with some evaluated in clinical trials (Zhu et al., 2018). The presence of branched-chain (methyl 8-methyl nonanoate) and polyacetylene derivatives of classical fatty acid esters (palmitate) suggests higher-order lipid metabolism. These compounds have been reported in previous studies to exert various health-related biological activities (Patel et al., 2022; Riecan et al., 2022). Fatty acid esters of hydroxy fatty acids have demonstrated anti-diabetes (Syed et al., 2019; Syed et al., 2018), anti-cancer (Rodríguez et al., 2019; Zhu et al., 2017), anti-inflammatory (Kolar et al., 2019), cardiovascular protective (Dongoran et al., 2020), and hepatoprotective activities (Benlebna et al., 2021; Defour et al., 2021) in mammals. The presence of methyl 8-methyl-nonanoate in *H. subaxillaris* may indicate similar roles in lipid metabolism modulation, which is worthy of investigating its ecological or pharmaceutical role. The phenolic antioxidant 3,5-di-tert-butylphenol may contribute to a protective role against oxidative stress, as reported in previous studies (Hoang and Park, 2024).

The phytochemical profile of *H. subaxillaris* is distinct due to its unique combination of sesquiterpenoid alcohols, polyacetylenic fatty acid esters, and phenolic antioxidants. This contrasts with the diterpenoid–phenol synergy observed in *C. getulus*, the monoterpenoid dominance of *A. spinosus*, and the simpler phenol ester profile of *V. sinuatum*. The relatively high geraniol content of *H. subaxillaris* may be consistent with insect-repellent functions reported for geraniol in previous studies, while its uncommon acetylenic compounds could potentially reflect ecological adaptations such as tolerance to arid habitats or defense against infection (Maeda, 2019; Tian et al., 2025). Among the extracts tested, *H. subaxillaris* showed the highest antibacterial activity in our bioassays. According to the literature, this effect may result from a combined action of terpenoids and polyacetylene-containing compounds, including their membrane-disruptive effects; however, this mechanistic explanation remains purely (Guo et al., 2017). Although A. spinosus displayed weaker antibacterial activity in our assays, its extract contains terpenoids and fatty acid esters that have been associated in the literature with hepatoprotective and anti-inflammatory effects (Yao and Liu, 2023). In *V. sinuatum*, the abundance of methyl palmitate and phenolic esters is consistent with its traditional use in respiratory conditions, and may contribute to the moderate antibacterial activity observed for the crude extract (Yabalak et al., 2022). In *C. getulus,* the combination of diterpenoids with antimicrobial phenolics suggests anti-inflammatory potential and possible synergistic effects against resistant pathogens (Kozyra et al., 2019). The chemically diverse profile of *H. subaxillaris*, characterized by both volatile defenses and lipid-based metabolites, underscores its pharmacogenetic value, while the remaining species demonstrate complementary bioactivities that need further investigation.

Quantitative phytochemical profiling revealed significant variation in the TPC and TFC among the four species. *H. subaxillaris* exhibited the highest TPC, comparable to *V. sinuatum* and *C. getulus*, and significantly greater than *A. spinosus*. Polyphenols, a broad class of secondary metabolites, play a central role in detoxifying hydrogen peroxide within plant cells and are integral to cellular antioxidant defense (Supritha and Radha, 2018). Beyond their role in plants, phenolic compounds are also critical to pharmacological action, functioning as antioxidants, metal chelators, and enzyme modulators (Sun and Shahrajabian, 2023). Their hydroxylated aromatic scaffolds enable efficient neutralization of free radicals, disruption of bacterial membranes, and inhibition of microbial enzyme systems (Veiko et al., 2020).

GC–MS analysis revealed several phenolic and terpenoid metabolites, and the high TPC observed in *H. subaxillaris* and *V. sinuatum* may be associated with their strong free radical scavenging capacity observed in the assays and may partially explain the antimicrobial activity of the crude extracts. Both species also contained elevated concentrations of flavonoids, a major subclass of phenolic compounds known for diverse bioactivities, including enzyme inhibition, antibacterial, anti-inflammatory, and antioxidant effects (Mutha et al., 2021). The planar structure of polyphenolic flavonoids favours interaction with bacterial nucleic acids and proteins, and thus enhanced antibacterial activity. Overall, plant polyphenols possess antimicrobial activity through numerous mechanisms involving proteins, DNA, cell walls, membranes, and energy metabolism (Lobiuc et al., 2023).

*C. getulus* possessed a moderate flavonoid content along with relatively high TPC and TFC levels. Its GC–MS profile, which is largely dominated by lipophilic terpenoids, suggests that non-phenolic lipophilic compounds may play an important role in the bioactivities reported for the crude extract. *A. spinosus,* on the other hand, contained the lowest levels of phenolics and flavonoids, consistent with its relatively low antioxidant and antimicrobial activity. This lower activity may reflect differences in phytochemical composition and abundance, potentially influenced by species-specific metabolic or ecophysiological traits, as suggested in previous studies. Despite its low TPC and TFC, A. spinosus contained elevated levels of monoterpenoid alcohols and fatty acid esters, which may indicate a relative shift toward volatile or lipid-based chemical defenses, as reported in the literature. Overall, these findings attest to the importance of both compound class and concentration in affecting biological activity (Rahman et al., 2019).

The relationship of phytochemical concentration and antimicrobial activity is complex and generally is synergistically based on combinations involving phenols, terpenoids, and fatty acid derivatives. Phenols and flavonoids, in turn, have a stronger relationship with antioxidant activity. The richness of phytochemicals reflected is an indication of the chemotaxonomic specificity of the individual species and their variegated uses in ethnopharmacological practice. The hydroxyl groups present in plant secondary metabolites have been widely reported to play a major role in their bioactive functionality, and hence these compounds are natural sources of paramount importance containing antioxidants (Abou Auda and Eleyan, 2025). In line with their high TPC and TFC values, *H. subaxillaris* exhibited the strongest free radical scavenging activity in the DPPH assay, followed by *V. sinuatum*. The latter contains phenolic compounds such as phenol and 2,4-bis(1,1-dimethylethyl)-, both of which demonstrate anticancer and free radical scavenging properties (Rahman et al., 2019).

The tert-butyl groups in 2,4-bis(1,1-dimethylethyl)- have been reported to play a major role in antioxidant activity in previous studies. These groups have been proposed to enhance the stability of the aromatic hydroxyl group by facilitating phenoxyl radical formation and hydrogen donation, thereby limiting lipid peroxidation (Zhao et al., 2020). This mechanistic interpretation is supported by previous *in silico* molecular docking studies, rather than by direct experimental evidence in the present work (Mostofa et al., 2024). *H. subaxillaris*, distinct polyacetylenes, including monoterpenoid alcohols, sesquiterpenoid alcohols, and falcarinol, have been shown to reduce oxidative stress and may also contribute to its antioxidant activity (Medbouhi et al., 2018; Tel-Çayan and Duru, 2019; Yu et al., 2024). Their potential prooxidant role is suggested by the synergistic interaction between citronellol and geraniol (Mostofa et al., 2024). By contrast, the comparatively lower TPC and TFC values in *A. spinosus* and *C. getulus* are consistent with their weaker antioxidant activities.

Hexane extracts of *H. subaxillaris*, *A. spinosus*, *V. sinuatum*, and *C. getulus* demonstrated measurable inhibitory activity against four clinically relevant bacterial strains: *B. cereus*, MRSA, *E. coli* O157:H7, and *P. aeruginosa*. Notably, *B. cereus* and MRSA were more susceptible to these lipophilic plant extracts than *E. coli* O157:H7 and *P. aeruginosa*, consistent with earlier reports that Gram-positive bacteria are generally more sensitive to plant-derived hydrophobic compounds (Aydın Kurç et al., 2023; Benramdane et al., 2022; Jubair et al., 2021; Palmeri et al., 2020).

Among the extracts, *H. subaxillaris* exhibited the strongest antibacterial activity across all tested strains, followed by *V. sinuatum*, *C. getulus*, and *A. spinosus*. While *H. subaxillaris* and *V. sinuatum* were active against both Gram-positive and Gram-negative bacteria, *C. getulus* and *A. spinosus* showed markedly weaker activity, particularly against *P. aeruginosa*, a pathogen characterized by multidrug resistance, biofilm formation, and active efflux mechanisms (Lorusso et al., 2022; Mirani et al., 2017; Tuon et al., 2022). The antibacterial activity of plant-derived phytochemicals is influenced by chain length, degree of unsaturation, and isomerism, with *cis*-isomers generally more potent than their trans counterparts (Alves et al., 2020). For instance, *cis*-6-hexadecenoic acid exerts inhibitory effects at low concentrations by disrupting the proton gradient, altering membrane fluidity, and impairing electron transport (Casillas-Vargas et al., 2021). Furthermore, esterified fatty acids tend to be more active against bacteria compared to their free acid forms, a feature that may be responsible for the efficacy of these extracts (Yoon et al., 2018).

The distinctive phytochemical profile of *H. subaxillaris* may contribute to its antibacterial action. Previous studies have reported that phenolic compounds can promote protein denaturation and oxidative stress in bacterial cells; polyunsaturated and branched-chain fatty acid esters may interfere with bacterial cell wall integrity and autolytic processes; and sesquiterpenoid and monoterpenoid alcohols have been associated with membrane perturbation and anti-inflammatory effects (Alves et al., 2020; Li et al., 2022; De Rossi et al., 2025).

These activities not only facilitate antimicrobial efficacy but also reduce the potential for development of resistance through synergistic interactions. *V. sinuatum* was also found to have notable antibacterial activity that was especially marked against MRSA and *B. cereus*. This activity may be related to its phytochemical composition, which includes alkaloids, phenolic antioxidants, and saturated fatty acid esters that have been reported to possess antibacterial properties in previous study (Ucan Turkmen et al., 2024). While these constituents share some antibacterial properties with those of *H. subaxillaris*, their disparate molecular structures may confer complementary or even independent mechanisms of action.

The potent activity of *H. subaxillaris* and *V. sinuatum* against *MRSA* and *B. cereus* may reflect synergistic interactions of phenolic antioxidants, terpenoid alcohols, and fatty acid esters. Multi-mechanism approaches like this one would also be expected to have less likelihood of resistance compared to single-agent therapies. This study used agar well diffusion as a preliminary screening method and future studies should focus on determining MIC and MBC values.

## Conclusion

This study presents a thorough phytochemical analysis of four Mediterranean medicinal plants (*V. sinuatum*, *A. spinosus*, *C. getulus*, and *H. subaxillaris*), and it yields an extensive array of lipophilic metabolites. Of all these, *H. subaxillaris* was the most antibacterial and antioxidant-active because of its distinctive set of sesquiterpenoids, polyacetylenic fatty acids, and phenolic antioxidants. *V. sinuatum* and *C. getulus* also demonstrated considerable activity due to their phenolic and diterpenoid profiles. The weak performance of *A. spinosus* underscores the role of phytochemical diversity in bioactivity. These findings underscore the value of phytochemical profiling for bioprospecting and pave the way for future pharmacognostic exploration of structurally diverse natural compounds from underexplored medicinal flora. Future work should isolate key metabolites (e.g., falcarinol, methyl 8-methyl-nonanoate) to mechanisms of action and elucidate structure-activity relationships.

## Data Availability

The original contributions presented in the study are included in the article/supplementary material, further inquiries can be directed to the corresponding author.
